# Healthy Eating Policy Improves Children’s Diet Quality in Early Care and Education in South Carolina

**DOI:** 10.3390/nu12061753

**Published:** 2020-06-11

**Authors:** Daniel A. Zaltz, Amelie A. Hecht, Roni A. Neff, Russell R. Pate, Brian Neelon, Jennifer R. O’Neill, Sara E. Benjamin-Neelon

**Affiliations:** 1 Department of Health, Behavior and Society, Johns Hopkins Bloomberg School of Public Health, 615 N Wolfe St, Baltimore, MD 21205, USA; sara.neelon@jhu.edu; 2 Department of Health Policy and Management, Johns Hopkins Bloomberg School of Public Health, 615 N Wolfe St, Baltimore, MD 21205, USA; ahecht3@jhu.edu; 3 Johns Hopkins Center for a Livable Future, Johns Hopkins Bloomberg School of Public Health 111 Market Pl, Suite 840, Baltimore, MD 21202, USA; rneff1@jhu.edu; 4 Department of Environmental Health & Engineering, Johns Hopkins Bloomberg School of Public Health, 615 N Wolfe St, Baltimore, MD 21205, USA; 5 Department of Exercise Science, University of South Carolina Arnold School of Public Health, 921 Assembly St, Columbia, SC 29208, USA; RPATE@mailbox.sc.edu (R.R.P.); ONEILLJR@mailbox.sc.edu (J.R.O.); 6 Division of Biostatistics, Department of Public Health Sciences, Medical University of South Carolina, 135 Cannon St, Charleston, SC 29415, USA; neelon@musc.edu

**Keywords:** policy, healthy eating, early care and education

## Abstract

Policies to promote healthy foods in early care and education (ECE) in the United States exist, but few have been prospectively evaluated. In South Carolina, a statewide program serving low-income children in ECE enacted new policies promoting healthy foods. We conducted an evaluation to measure changes in dietary intake among children in ECE exposed and not exposed to the new policy. Using direct observation, we assessed dietary intake in 112 children from 34 ECE centers in South Carolina and 90 children from 30 ECE centers in North Carolina (a state with no policy). We calculated Healthy Eating Index-2015 (HEI) scores to measure diet quality consumed before and after the policy was enacted. We fit mixed-effects linear models to estimate differences in HEI scores by state from baseline to post-policy, adjusting for child race, number of children enrolled, director education, center years in operation, participation in the Child and Adult Care Food Program (CACFP), and center profit status. The policy increased HEI scores for whole fruits, total fruits, and lean proteins, but decreased scores for dairy. Thus, the policy was associated with some enhancements in dietary intake, but additional support may help improve other components of diet.

## 1. Introduction

The global prevalence of childhood overweight and obesity significantly increased over the past four decades [1], contributing to vast social, economic, and health burdens [2]. Children with overweight or obesity are at an increased risk of chronic conditions, including high blood pressure [3], asthma [4], and type 2 diabetes [5], as well as persistent obesity [6], stigma and related mental health outcomes [7], and cardiovascular diseases in adulthood [8,9]. Early childhood is a critical intervention period to prevent obesity and reduce potential negative health impacts later in life [10].

Policies that promote healthy eating have the ability to reach broad audiences and influence obesogenic behaviors with relatively low resource utilization [11], and thus play a central role in global childhood obesity prevention [12]. The early care and education setting (ECE) is one such venue to implement these policies [13], given that the majority of children ages 3 to 5 years in most Western countries regularly attend ECE, where they are likely to receive a significant portion of their daily meals and snacks [14,15]. There is some evidence that children attending ECE have a higher prevalence of obesity than others [16,17,18]. Moreover, children may receive inadequate servings of fruits, vegetables, and whole grains in ECE, as well as excessive amounts of saturated fats and added sugars [19,20,21,22]. Policies designed to improve diet quality in ECE may have long term benefits, since dietary intake during early childhood impacts eating habits and health outcomes throughout life [23,24,25].

Dietary intake during early childhood and beyond is impacted by complex intersections of individual behavior factors, like food preferences [26], and broader cultural, environmental, and socioeconomic factors, including race, ethnicity, gender, and education [27,28,29]. As such, healthy eating policies in ECE can take on many forms depending on their targeted outcome, which may include changes to child or provider eating behaviors, the food environment, mealtime practices, or knowledge and resources needed to provide healthier foods [13,30]. Differences in how governments regulate and administer child care also contribute to the heterogeneity in the design and implementation of healthy eating policies in ECE [31,32,33]. Given this heterogeneity, evaluations and subsequent literature reviews have been unable to identify strong, generalizable evidence to improve policy design and implementation [30,34,35]. This is partially because a significant proportion of this evidence focuses on local pilot programs rather than broader policies, which may be more difficult to evaluate due to resource constraints or challenges with measuring changes over time [30,34,35]. Researchers, experts, and stakeholders have correspondingly called for higher-quality evaluations of healthy eating policies in ECE [32,36], with a specific focus on the importance of natural experiments to evaluate policy impacts over time [37].

Therefore, the purpose of this study was to evaluate the impact of a state-level healthy eating policy on the diet quality of children in ECE. The study took place in South Carolina (SC) in the United States (USA). We used North Carolina (NC), a bordering state with no contemporaneous policy change, as the comparison. We hypothesized that children in ECE centers subject to the policy would improve overall and specific components of their diet quality, with no corresponding improvement in children in ECE centers in the comparison state.

## 2. Materials and Methods

### 2.1. Overview

This study analyzed the dietary intake of preschool-aged children in South Carolina ECE centers before and after the implementation of a healthy eating policy. We conducted a two-group pretest/post-test study and used North Carolina, where no policy changes occurred, as the comparison. We collected baseline and follow-up data in the same centers but not necessarily among the same children. Centers were eligible for participation in this study if they received state subsidies for providing care to low-income children through a statewide program and had no open case of abuse or neglect with the state licensing agency. We mailed recruitment letters to all eligible ECE directors (174 in SC, 168 in NC), and enrolled the first 30 who responded from each state. The research team enrolled an additional four centers in South Carolina to accommodate a high interest in participating among center directors. We obtained written, informed consent from ECE center directors and notified all parents of study procedures prior to collecting data. We did not collect any identifiable information related to individual children. The Institutional Review Boards of the University of South Carolina (#00014606) and Duke University (#00033793) approved this study, both in 2012.

### 2.2. Policy in South Carolina

The policy was implemented in April 2012 in South Carolina ECE centers by the Department of Social Services through a quality rating and improvement system (QRIS) called ABC Quality [38]. All participating centers were required to follow this new policy by October 2012 as part of their licensing requirements. The policy was designed to improve nutrition-related health outcomes for children ages 5 years and younger. The design and implementation of this policy included expert and stakeholder input from researchers, policymakers, and ECE providers, and has been described in detail elsewhere [39]. Centers were required to serve specific foods and beverages to children to comply with the policy. Children aged two years and older were required to be served skim or 1% milk only; one four-ounce serving of 100% fruit juice per day or water, two whole fruits, at least one vegetable (not including white potatoes), and at least one serving of whole grains. High-fat meats and sweet food items (e.g., cookies, cakes) were limited to two servings per week, and fried vegetables were limited to one serving per week. Additionally, ECE staff were required to attend annual nutrition trainings, implement age-appropriate nutrition lessons at least weekly in the classroom, and were prohibited from using food as a reward or punishment. All participating centers also needed to create and implement a written nutrition policy.

### 2.3. Outcome

We collected baseline dietary intake data during the two months preceding the policy implementation and then three months after its implementation, using the previously-validated Diet Observation in Child Care methodology [40,41]. Details of this method are available elsewhere [40,41,42,43]. Briefly, we trained and certified data collectors using pre-specified inter-rater reliability requirements, who then recorded all foods and beverages served to and eaten by children in each participating ECE center. Data collectors randomly selected up to three children in one classroom per center to observe throughout one day of regular care. Following pre-specified protocol, data collectors counted off all children present in the classroom and chose the first, third, and fifth child, with the seventh, if present, as an alternate [41]. We did not necessarily collect dietary intake data from the same children at baseline and follow-up since we were primarily interested in center-level dietary intake. Data collectors recorded all foods and beverages served to and consumed by children with as much detail as possible, including foods traded, discarded, and remaining at the end of all meals and snacks. The data collectors do not interact with children during the observations and are trained to discretely monitor selected children [41]. During observations, data collectors record distinguishing characteristics (e.g., “blue shirt”) on collection forms to easily identify selected children and situate themselves in the classroom in such a way that all children may be observed simultaneously [41]. Data collectors recorded all recipes of foods prepared onsite and brand names of pre-packaged foods and beverages. The research team inputted these data into the Nutrition Database for Scientific Research version 2012 (Nutrition Coordination Center, University of Minnesota, Minneapolis, MN, USA) [44]. A trained researcher reviewed all dietary records for completeness and accuracy prior to exporting files for further analyses.

To assess diet quality, we calculated Healthy Eating Index (HEI) scores for all foods and beverages consumed by children in centers. We used the most recent scoring standards, updated in 2015 [45]. The HEI is a calorie-adjusted comparison of individual or group-level dietary intake to current dietary guidelines [46]. The HEI score was updated in 2015 and represents the sum of thirteen component scores. Higher scores are awarded for healthier intake within each component. The HEI components and maximum scores include total fruit (5), whole fruit (5), total vegetables (5), greens and beans (5), whole grains (10), dairy (10), total proteins (5), seafood/plant (lean) proteins (5), fatty acids (10), refined grains (10), sodium (10), added sugars (10), and saturated fats (10). To calculate these component scores, dietary data is expressed as a ratio per 1000 kilocalories, except for the fatty acids component, which is calculated as a ratio of polyunsaturated and monounsaturated fatty acids to saturated fatty acids. Higher consumption of food items within each component receives a higher score except for refined grains, sodium, added sugars, and saturated fats, which receive higher scores for lower intake. We applied the population ratio method to calculate and compare HEI scores between the two states, which utilizes mean dietary intake data and calculates scores at the group-level [46]. The population ratio method is recommended to calculate HEI scores at the population or sub-population level and to compare differences between groups. We utilized this method because we were interested in overall diet quality in ECE centers before and after the implementation of the policy.

### 2.4. Other Measures

During baseline data collection, directors from each ECE center completed a brief survey related to center characteristics. Directors reported total enrollment by age, number of income-subsidized children, total years in operation, number of teachers and classrooms, participation status in the Child and Adult Care Food Program (CACFP), profit status (for profit vs. non-profit), and child race and ethnicity as a percent of total enrollees. Directors also reported their highest level of education, and age, race, ethnicity, and gender.

### 2.5. Analysis

We calculated means, standards deviations, and proportions to summarize center characteristics. To evaluate the impact of the policy, we fit mixed-effects linear regression models [47] to estimate the difference, by state, in the change from baseline to post-policy HEI scores of foods consumed (primary outcome) and HEI component scores (secondary outcome). To calculate HEI scores from the Nutrition Database for Scientific Research output data, we implemented SAS software (SAS Institute Inc., Cary, NC, USA) scripts developed by the Nutrition Coordinating Center at the University of Minnesota [48] using R Statistical Software version 3.5.1 (R Foundation for Statistical Computing, Vienna, Austria). We included indicators for time, state, and an interaction between time and state (difference-in-difference estimator) in all models. We conducted unadjusted analyses and adjusted analyses using fixed effects for covariates identified a priori based on prior literature (child race, number of children enrolled, director education, center years in operation, participation in CACFP, and profit status) [49,50,51]. Due to missing covariate data, two centers were excluded from adjusted analyses. We included center random effects and robust standard errors in all models. We assessed the potential influence of extreme cases using multiple metrics (e.g., Cook’s D, studentized residuals) and conducted sensitivity analyses excluding influential cases. We conducted these analyses using Stata/IC version 14.1 (StataCorp LLC, College Station, TX, USA) and a significance level of *p* < 0.05.

## 3. Results

We observed 102 children from 34 centers in South Carolina and 90 children from 30 centers in North Carolina at baseline. At follow up, we observed 99 children at 33 centers in South Carolina and 78 children at 26 centers in North Carolina; five centers closed or withdrew during the study period (four in North Carolina, one in South Carolina). Centers lost to follow-up were demographically similar to those included at follow-up (results not shown). At baseline, South Carolina ECE centers enrolled more 3- to 5-year-old children than North Carolina ECE centers (mean (SD) 46.0 (34.1) vs. 25.4 (23.1)), and a lower mean (SD) percentage of Hispanic/Latino(a) children (1.4% (2.6) vs. 4.8% (7.3)) (Table 1). More directors from South Carolina reported receiving a four-year degree or higher, compared to directors from North Carolina (72.7% vs. 60.0%), and fewer South Carolina ECE centers were for-profit, compared to North Carolina ECE centers (86.7% vs. 52.9%). There were no other significant differences in baseline demographic characteristics between the two states. These differences in demographic characteristics were detected in comparisons of North Carolina and South Carolina centers included at baseline and included at follow-up. Comparing centers included at follow-up, one additional difference emerged: South Carolina ECE centers had a higher mean percentage of Black children compared to North Carolina centers (at baseline: 60.5% vs. 42.6%, *p* = 0.06; at follow-up: 61.5% vs. 41.8%, *p* = 0.05).

At baseline, children had a mean (SD) HEI of 56.0 (11.5) in South Carolina and 55.9 (10.2) in North Carolina, out of 100 possible points (Table 2). At follow-up, South Carolina children increased HEI scores by 4.3 points to 60.3, whereas North Carolina scores increased by 2.0 points to 57.8. After adjustment, there was a non-significant increase in total HEI score of 3.1 points (95%CI: −4.2, 10.5; *p* = 0.41) in South Carolina compared to North Carolina (Table 2). However, children’s HEI component scores in South Carolina increased significantly for total fruits (0.8 points; 95%CI: 0.2, 1.5; *p* = 0.01), whole fruits (0.9 points; 95%CI: 0.06, 1.8; *p* = 0.04), and seafood/plant proteins (1.2 points; 95%CI: 0.06, 2.3; *p* = 0.04), compared to children in North Carolina. Children in South Carolina scored lower on the HEI dairy component score after the implementation of the policy, for an overall adjusted difference-in-difference of −1.3 points (95%CI: −2.3, −0.3; *p* = 0.01). There were no other significant differences in HEI component scores between the two groups.

Findings were similar in magnitude and statistical significance across unadjusted and adjusted analyses. (Table A1) We did not identify any extreme values and findings were robust to sensitivity analyses excluding influential cases (results not shown).

## 4. Discussion

In this prospective evaluation of a healthy eating policy targeting low-income children in ECE in South Carolina, we found that children’s overall diet quality did not improve significantly three months after the policy. However, children in centers subject to the policy improved their intake of fruit and lean proteins, compared to children in centers with no such policy. The healthy eating policy specifically targeted fruit and lean protein consumption, and thus these changes are in the anticipated direction.

The finding that total HEI scores (overall diet quality) did not improve is not expected. Overall HEI scores in children in both states were relatively low (about 60 out of 100 possible points) but consistent with prior observations of children’s dietary intake in ECE centers [21,52,53,54] and family child care homes [55,56]. In these studies, consistent with our findings, children scored highest in whole fruit, total fruit, and dairy components. These overall findings reiterate the need to improve children’s dietary intake in ECE, with a focus on other dietary components like vegetables and whole grains—components for which we did not see improvement.

Children in ECE centers subject to the policy significantly improved their intake of total and whole fruit. Increasing fruit consumption among young children is an important public health goal since many do not currently receive adequate servings of fruit [57], and those who do may be less likely to become overweight or obese [58] and have decreased all-cause mortality across the lifespan [59]. Pooled results from prospective cohort studies show this relationship to reach its peak at five daily servings of fruits and vegetables [59], which is consistent with USA and European Union dietary guidelines for children [60,61]. In this study, we observed total and whole fruit component scores at or above four out of five possible points, which is consistent with prior evidence of children’s fruit consumption in ECE [21,52,53,54,56]. There is additional evidence that children consume more fruit in ECE than at home [62], which may be partially explained by the broader observation that children exhibit less picky eating behaviors in ECE versus at home [63]. The ECE environment may thus provide an important venue to expose children to a variety of foods and improve and maintain a healthy consumption of fruit among young children. Healthy eating policies that encourage taste tests and exposure to a variety of fruits may increase consumption in ECE, but more research is needed to better understand this relationship [35].

Children in centers subject to the policy also improved their consumption of seafood and plant proteins. There is reason to focus on protein sources, rather than protein as a single macronutrient, in young children’s diets. First, the transition from infancy to toddlerhood is associated with a diversification of nutrient sources, during which many plant and animal foods replace formula or breastmilk as primary sources of protein [64]. There is longitudinal evidence of an association between protein sources and adiposity among children ages birth to 5 years, with collective results suggesting that lean proteins, particularly vegetable proteins, may be protective against excess body weight [65,66]. Protein sources are thus an important target of healthy eating policies in ECE, in light of prior evidence that children in these settings do not consume adequate amounts of lean proteins [52,54,56]. Although lean protein consumption improved in ECE centers that implemented the policy, overall scores remained low, reaching a maximum of only 1.3 out of 5 possible points. Importantly, component scores for lean proteins were lower than scores for total proteins. There is therefore room to improve sources of proteins in foods served to children in ECE. Moreover, healthy eating policies in ECE should seek to align with best practice recommendations to reduce consumption of high-fat meats like pre-fried nuggets [67]. Replacing high-fat meats with leaner options may provide an additional micronutrient benefit since lean proteins are typically richer in zinc, iron, and potassium, all of which tend to be consumed in inadequate amounts among young children [64].

We found that HEI component scores for dairy consumption decreased among children in intervention centers. Overall, dairy HEI scores remained high (>9/10) among children in intervention and comparison states before and after policy implementation, and prior studies have similarly reported high dairy HEI component scores among children in ECE [21,52,53,54,56]. The dairy component score included all fluid milk, cheese, and yogurt, but prior research suggests that young children in ECE receive most of their dairy intake from fluid milk [68]. Milk is a primary source of total caloric intake among young children [69], and recent studies showed most children consumed plain, unflavored, reduced-fat (2%), or low-fat (1%) milk in ECE [53,62,70]. This slight decrease in HEI component score may be partially attributable to decreases in servings of milk in South Carolina centers, possibly in response to nutrition rules that required serving only low- or non-fat milk. It is also possible that children require more time to shift to changes in what type of milk they are served in ECE. Still, replacing full or reduced-fat (2%) milk with low or non-fat milk still provides children with necessary nutrients, like calcium, vitamin D, and potassium, but without excess calories and fat [71].

There are a variety of data collection methods and research designs used to evaluate healthy eating policies in ECE. In an evaluation of a statewide healthy eating policy in Delaware (USA), researchers found significant variation in compliance (but not child dietary intake) with different dietary components, with the majority of centers complying with juice regulations but less than one out of five complying with whole-grain requirements [72]. Identifying patterns and predictors of compliance with healthy eating policies in ECE may improve future policy design, but cannot measure policy impacts over time [73]. Other studies have described associations between ECE policies and child outcomes using retrospective methods, but these too are unable to assess changes over time. For example, in 2018, researchers evaluated correlations between local- and state-level childhood healthy eating and physical activity policies and obesity rates [74] and found that the 4 pre-selected study locations with declining childhood obesity rates had contemporaneously enacted relevant policies [75]. This scoping retrospective described general relationships between the policy environment and childhood obesity, but could not, as the authors noted, elucidate causal pathways [75]. Cross-sectional or retrospective evaluations may yield rich quantitative and qualitative data about healthy eating in ECE [76], but changes over time cannot be assessed and thus policy impacts remain unmeasured.

Several studies have addressed this gap and prospectively measured changes to children’s diet before and after the implementation of new policies in ECE. In California (USA), for example, researchers administered comprehensive surveys to a random sample of ECE directors before and after the implementation of the healthy beverage policy [77]. Results from this prior evaluation showed significant improvements to the beverages served in ECE centers after the implementation of a policy [77], with a specific increase in the availability of water, which was a direct policy target [78]. The results from our study exceed the scope of prior prospective evaluations by collecting directly observed dietary intake data and including a comparison group to control for secular changes, which may be particularly important when studying natural experiments of policy changes in ECE. One recent study evaluating a new statewide physical activity policy in ECE found significant improvements in children’s physical activity within the intervention state, but could not link these changes to the new policy, given similar improvements over time in a bordering state [79]. Considering its data collection methods and design, our study represents one of the most rigorous evaluations of a state healthy eating policy in ECE.

Our study also has limitations. First, we evaluated a natural experiment and thus were not able to randomize states to intervention (policy) or control. It is possible that there were differences between the two states that could account for some of our findings. Additionally, North Carolina could have made changes unbeknownst to our study team that influenced our results. However, given that we were not able to randomize, our quasi-experimental research design is recommended to account for secular changes when assessing the impact of a nutrition policy on dietary quality [37]. Next, five sites, including four in North Carolina and one in South Carolina were lost to follow-up; these sites, however, were demographically similar to those not lost to follow-up. Additionally, our dietary assessment method has limitations. We did not conduct plate waste studies, which have been used to describe diet quality in ECE [80] and provide researchers with a more precise estimate of dietary intake versus direct observation methods [81]. However, our observation method has been tested for both reliability and validity [41] and is considered a robust measurement technique in ECE [36]. Moreover, researchers often rely on survey or self-reported data from parents and providers to assess changes in children’s dietary intake [82], which are useful for some research purposes [83] but do not yield levels of detail and accuracy gained from more other methods like weighing and measuring foods or directly observing meals and snacks in ECE [36,40]. We evaluated children’s dietary intake via direct observation methods, which provide details and reductions in social desirability bias common in self-report survey data [84]. These methods are less common, and few evaluations of healthy eating policies have employed them [82], likely because of their associated costs and high staffing burden [36].

We did not necessarily collect dietary intake data from the same children in each center during baseline and follow-up and therefore could not account for the potential impact of picky eating or food neophobia. However, in a prior study, fewer than 20% of center directors in South Carolina reported children’s taste preferences as a barrier to implementing the policy [85]. In the same study, directors reported cost as the most prevalent barrier [85], which adds to evidence that resource and environmental barriers are often reported during implementation studies of healthy eating policies in ECE [86]. Still, it is important to note that more than half of all directors who implemented this policy in South Carolina reported perceptions of being well-informed about the policy components and their likely impact on day-to-day changes in ECE [85]. Future studies should continue to assess resource barriers to implementing new healthy eating policies in ECE, which may ultimately impact intended policy effects [34]. We also conducted follow-up assessments relatively soon after the policy was implemented. Due to limited funding, we were not able to assess the longer-term implications of the policy. Finally, ours was a relatively small convenience sample of children and centers, which limits the generalizability of our findings. Despite this, our study is one of the first to prospectively evaluate a new statewide healthy eating policy in ECE, employing more robust data collection methods and a comparison group. Future studies could include larger numbers of centers and children to increase the generalizability of findings.

## 5. Conclusions

Children exposed to the policy in South Carolina exhibited modest improvements in some components of their diets, but the policy did not appear to impact overall diet quality as measured by the HEI. Healthy eating policies in ECE have the potential to improve children’s diet quality and may be an important intervention to improve health and reduce childhood obesity. Future research should assess the impacts of healthy eating policies in the longer term, making use of larger sample sizes and longer follow-up periods.

## Figures and Tables

**Table 1:** Baseline characteristics of the 64 ECE centers in the policy evaluation study by the state in 2012.

	South Carolina (n = 34)	North Carolina (n = 30)
Center characteristic	Mean (SD)	
Children Enrolled, 3–5 Years Old	46.0 (34.1)	25.4 (23.1)
Subsidized Children	26.5 (31.3)	19.7 (17.6)
Years in Operation	18.2 (12.9)	18.3 (17.1)
Number of Teachers	10.9 (6.9)	9.8 (9.4)
Number of Classrooms	6.2 (3.1)	5.3 (3.0)
	% (N)	
For-Profit Status	52.9 (18)	86.7 (26)
	Mean (SD)	
Child Race/Ethnicity		
% Black/African American	60.5 (40.8)	42.6 (32.6)
% Asian/Pacific Islander	0.3 (0.6)	4.6 (15.1)
% White	32.1 (37.3)	39.9 (32.8)
% Hispanic/Latino(a)	1.4 (2.6)	4.8 (7.3)
% Multiple Races	5.0 (17.4)	6.9 (11.7)
% Native American	0.03 (0.2)	0.03 (0.2)
% Other Race	0.03 (0.2)	0.03 (0.2)
	% (N)	
Director education		
High school/Community College	27.3 (9)	40.0 (12)
Some or all 4-Year College	33.3 (11)	53.3 (16)
More Than 4-Year College	39.4 (13)	6.7 (2)

**Table 2:** Adjusted ^a^ differences ^b^ in Healthy Eating Index (HEI) total and component scores in children in South Carolina ECE centers post-policy, compared to North Carolina (n = 62).

	South Carolina (Policy)		North Carolina (Comparison)				
Healthy Eating Index (Max Score)	Baseline	Follow-Up	Baseline	Follow-Up	Difference-in- Difference	*p* Value	95%CI
Adequacy Components							
Total Score (100)	56.0	60.3	55.8	57.8	3.1	0.41	−4.2, 10.5
Total Fruits (5)	3.9	4.5	4.6	4.4	0.8	0.01	0.2, 1.5
Whole Fruits (5)	4.3	4.5	4.6	4.0	0.9	0.04	0.06, 1.8
Total Vegetables (5)	2.1	1.9	2.0	1.5	0.1	0.84	−0.8, 1.0
Greens and Beans (5)	1.4	1.2	0.9	0.6	−0.2	0.77	−1.4, 1.1
Whole Grains (10)	2.4	3.2	2.2	3.1	0.1	0.91	−2.1, 2.4
Dairy (10)	9.9	9.1	9.4	9.9	−1.3	0.01	−2.3, −0.3
Total Protein Foods (5)	3.0	3.2	3.6	2.7	1.1	0.11	−0.3, 2.4
Seafood & Plant Proteins (5)	1.1	1.3	1.3	0.2	1.2	0.04	0.06, 2.3
Fatty Acids (10)	1.9	4.1	3.0	4.5	0.9	0.42	−1.3, 3.03
Moderation Components							
Refined Grains (10)	6.7	6.0	5.3	5.4	−0.7	0.55	−2.9, 1.5
Sodium (10)	5.2	5.3	4.6	5.8	−0.7	0.55	−3.1, 1.7
Added Sugars (10)	8.0	8.1	7.9	8.2	−0.1	0.94	−2.0, 1.9
Saturated Fats (10)	6.1	7.7	6.3	7.4	0.9	0.43	−1.3, 3.1

^a^ Adjusted for child race, number of children 3–5 years enrolled, director education, number of years center has been in operation, participation in the Child and Adult Care Food Program, and center profit status. ^b^ Models account for clustering within centers by clustering standard errors at the center level. Results are reported as predicted probabilities and marginal effects.

## Appendix A

**Table A1:** Unadjusted (n = 64) and adjusted ^a^ (n = 62) differences ^b^ in Healthy Eating Index (HEI) total and component scores in children in South Carolina ECE centers post-policy, compared to North Carolina.

	Unadjusted		Adjusted		
Healthy Eating Index (Max Score)	Difference-in-Difference	*p* Value	Difference-in- Difference	*p* Value	95% CI
*Adequacy Components*					
Total Score (100)	2.4	0.52	3.1	0.41	−4.2, 10.5
Total Fruits (5)	0.8	0.02	0.8	0.01	0.2, 1.5
Whole Fruits (5)	0.9	0.03	0.9	0.04	0.06, 1.8
Total Vegetables (5)	0.2	0.58	0.1	0.84	−0.8, 1.0
Greens and Beans (5)	0.03	0.96	−0.2	0.77	−1.4, 1.1
Whole Grains (10)	−0.06	0.96	0.1	0.91	−2.1, 2.4
Dairy (10)	−1.3	0.01	−1.3	0.01	−2.3, -0.3
Total Protein Foods (5)	1.1	0.12	1.1	0.11	-0.3, 2.4
Seafood & Plant Proteins (5)	1.3	0.02	1.2	0.04	0.06, 2.3
Fatty Acids (10)	0.70	0.52	0.9	0.42	−1.3, 3.03
*Moderation Components*					
Refined Grains (10)	−0.75	0.50	−0.7	0.55	−2.9, 1.5
Sodium (10)	−1.04	0.39	−0.7	0.55	−3.1, 1.7
Added Sugars (10)	−0.18	0.85	−0.1	0.94	−2.0, 1.9
Saturated Fats (10)	0.57	0.62	0.9	0.43	−1.3, 3.1

^a^ Adjusted for child race, number of children 3–5 years enrolled, director education, number of years center has been in operation, participation in the Child and Adult Care Food Program, and center profit status. ^b^ Models account for clustering within centers by clustering standard errors at the center level. Results are reported as predicted probabilities and marginal effects.
